# Vessel Occlusion, Penumbra, and Reperfusion – Translating Theory to Practice

**DOI:** 10.3389/fneur.2014.00194

**Published:** 2014-09-30

**Authors:** Bruce C. V. Campbell, Geoffrey A. Donnan, Stephen M. Davis

**Affiliations:** ^1^Department of Medicine, Royal Melbourne Hospital, University of Melbourne, Parkville, VIC, Australia; ^2^Florey Institute of Neuroscience and Mental Health, University of Melbourne, Parkville, VIC, Australia

**Keywords:** stroke, reperfusion, recanalization, thrombolysis, collateral flow, perfusion imaging

The management of ischemic stroke is at a critical juncture. Administration of intravenous tPA is currently restricted to within 4.5 h from stroke onset with several trials in longer time windows proving neutral (1, 2). Revascularization success with tPA in major vessel occlusion is widely recognized as suboptimal (3). Alternative thrombolytic agents with theoretical efficacy advantages such as tenecteplase and desmoteplase are yet to show benefit in phase 3 trials. The promise of endovascular therapy has also yet to translate into positive randomized trials (4–6), although a new generation of devices is currently being studied. While it is possible that these therapeutic approaches are simply ineffective, the heterogeneity of stroke pathophysiology is likely to be contributing to the neutral results we often observe.

Imaging selection has been proposed as a means of reducing heterogeneity by identifying patients with the potential to benefit from revascularization and therefore enhancing the probability of success in trials of new therapies. However, whether it is sufficient to demonstrate an occluded artery as the target or to also require evidence of salvageable downstream tissue has been debated. The recent announcement of neutral results in DIAS 3 (7), a trial that compared desmoteplase versus placebo 3–9 h after stroke onset in patients with vessel occlusion, without reference to downstream tissue status other than what was visible on non-contrast CT, will no doubt further stimulate this discussion. It is, therefore, salient to consider the current methods to identify salvageable ischemic penumbra and the potential value of commonly used surrogates for clinical outcome, chiefly reperfusion, recanalization, and infarct growth.

## Identifying Salvageable Tissue

There are some stroke patients who do not have an identifiable vessel occlusion. It is well recognized that such patients generally have an excellent natural history and will not benefit from revascularization therapy. This has led to one body of opinion that identifying vessel occlusion is the key criterion for treatment selection (8). It is true that the majority of patients with a vessel occlusion have some non-functioning but potentially salvageable ischemic penumbra downstream, at least early after stroke onset. However, patients with a large ischemic core at admission imaging not only have very little chance of benefit from treatment, they may well have worse outcome after “successful” revascularization due to hemorrhage and malignant edema and so actively detract from any positive treatment effect (9, 10). Unfortunately, in trials, there is also a risk of such patients being over-represented due to perceived lack of equipoise in those with more favorable imaging profiles or financial incentives to recruit.

There are several potential methods to rapidly identify salvageable tissue in clinical practice. In the absence of recanalization, collateral blood flow is the determinant of penumbral survival. Collaterals can be imaged using non-invasive CT or MR angiography (11, 12). Traditional static CT or MR angiography is limited in its ability to assess collateral flow as it is delayed (whereas CTA is timed to normal peak arterial flow) and relatively low flow (which reduces detection by time of flight MRA) leading to potential underestimation of collateral quality. However, dynamic acquisitions are now available for both CT and MR, including reformatted perfusion imaging protocols, and can fully characterize collateral flow (11, 13). These angiographic methods are typically scored using simple visual scales.

Perfusion imaging with CT or MR also provides a dynamic assessment of collateral flow with high temporal resolution and post-processing to represent delay and flow in a more quantitative manner. The perfusion maps require thresholding in order to separate potentially at risk “penumbra” from non-threatened “benign oligemia” as the visual extent of the abnormality overestimates tissue at risk (14, 15). For MR and CT, *T*_max_ (time to maximum) >6 s has been supported by several studies (16–19). When CT perfusion is used, a separate threshold to distinguish irreversibly injured “ischemic core” versus penumbra is required with cerebral blood flow being more accurate than cerebral blood volume for this purpose (20–22). The larger the “mismatch” between small core and large penumbra, the more likely it is that the patient will respond favorably to revascularization. Whichever method is chosen, better collateral flow scores and mismatch volumes are strongly and consistently associated with improved outcome after reperfusion. With the advent of fully automated perfusion processing software (23, 24), the argument that perfusion imaging is too complex, time consuming, or challenging to implement and standardize across multiple centers has become obsolete. Indeed, the objective reproducibility of “mismatch,” in contrast to visual scoring of collaterals, is a major advantage. The neutral DIAS 3 results with suggestion of benefit in the “per protocol” population (7) indicate that even accurately determining if there is a vessel occlusion poses challenges in a multicenter trial. Presumably, attempts to score collaterals, a much more subjective process, will require significant site education and training if such approaches are to be successful.

The alternative to directly visualizing collateral flow is to identify patients with large ischemic core, which is a direct result of poor collateral flow. Large ischemic core at admission imaging is a reliable indicator of poor outcome (25), although the location of the core also requires consideration. Diffusion MRI is the most accurate method of assessing core in current clinical practice (26). Major reversibility of diffusion lesions with currently available treatments appears uncommon, even in early time windows (27, 28). Thresholded cerebral blood flow or cerebral blood volume can generally provide similar information using CT perfusion imaging (20, 21, 29, 30).

It is important to remember that collateral flow in ischemic stroke is a dynamic process. The fluctuations in clinical severity that clinicians observe may result from fluctuation in collateral flow and, therefore, the snapshot provided by imaging may not reflect the collateral status that has been present over the entire period since stroke onset. This can lead to classification errors in both directions. An improvement in collateral flow can elevate CBF and CBV above the threshold for “core” and may cause temporary post-reperfusion reversal of the diffusion lesion leading to underestimation of core volume (27). A patient imaged just after a deterioration in collateral flow may appear to have a large core based on CBV or CBF and may even have a diffusion lesion but rapid recovery in collaterals could reverse this situation. However, it is important to realize that such cases are exceptions rather than the norm and do not negate the value of advanced imaging. Correlation with the clinical features can prevent misinterpretation in some cases.

## Reperfusion Versus Recanalization

The question of the most appropriate revascularization endpoint has been often debated (31). Early endovascular trials were criticized for assessing recanalization of the target vessel without consideration of downstream reperfusion. Clearly, opening the M1 segment of the middle cerebral artery without also establishing flow in M2 vessels is of little clinical value. This, however, reflects a flaw in the measurement scales rather than the concept of recanalization.

A significant advance has been the development of consensus around assessment of angiographic reperfusion that focuses on re-establishment of downstream perfusion with the “modified Treatment In Cerebral Ischemia” (mTICI) (32) score. There has been increasing recognition that a score of 2a (<50% reperfusion of the affected territory), which was included as a “successful” endovascular outcome in earlier studies does not lead to acceptable rates of good outcome. Even mTICI 2b (>50% reperfusion of affected territory) has significantly worse outcomes than mTICI 3 (complete reperfusion), emphasizing the importance of obtaining as close to full reperfusion as possible (4, 17).

**Figure 1:**
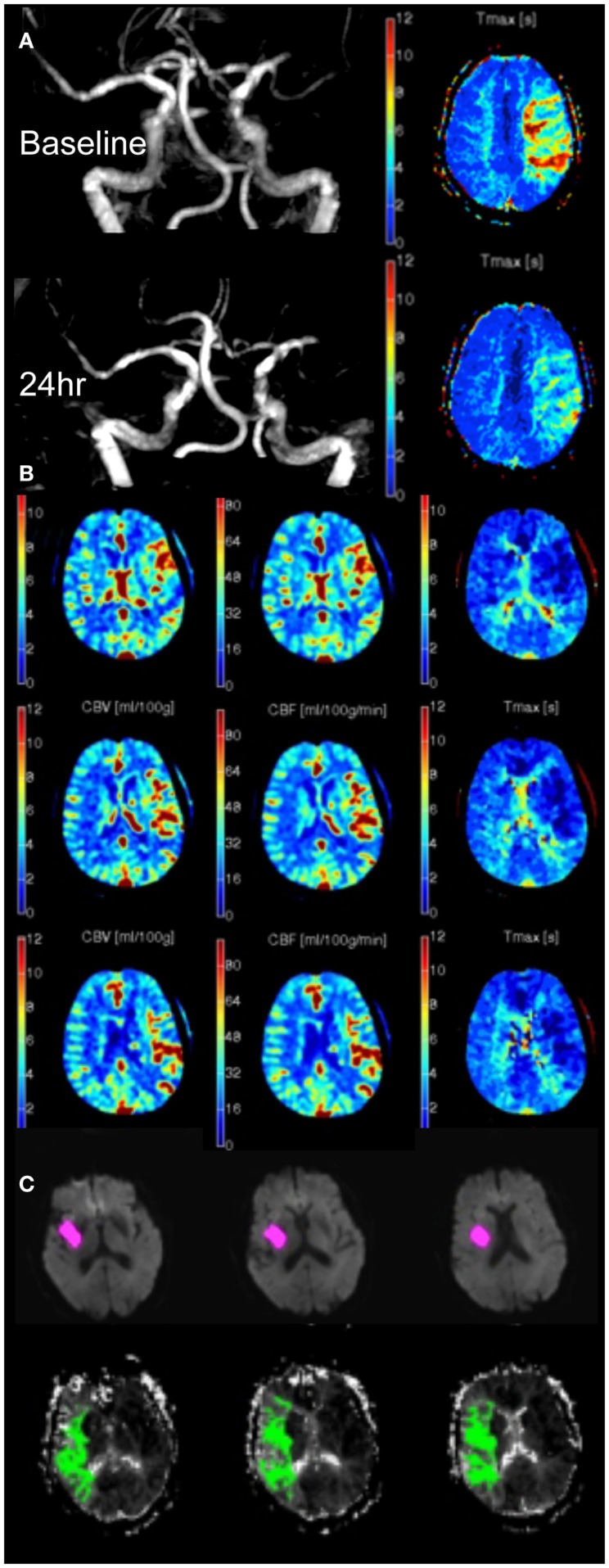
**(A)** MRA and *T*_max_ in a patient with left MCA occlusion at 5 and 24 h. Despite persistent occlusion, the volume of *T*_max_ > 6 s has reduced from 56 to 0 mL due to improved collateral supply. **(B)** Post-reperfusion hyperperfusion indicated by increased CBV (left), CBF (middle), and reduced *T*_max_ (right) in a patient with recent reperfusion of left MCA occlusion. **(C)** MRI diffusion (pink) and perfusion (*T*_max_ > 6 s, green) imaging 24 h post-stroke onset showing persistent hypoperfused tissue, which was contributing to the patient’s clinical deficit but had not developed diffusion restriction.

In general, recanalization of the major vessels does translate to tissue reperfusion. There are, however, scenarios where recanalization and reperfusion are incongruent, which are worthy of consideration. Recanalization can occur without reperfusion. As mentioned above, many descriptions of this in the literature relate to overly simplistic recanalization scales that focus too narrowly on one segment of the vascular tree without regard for the adjacent segments. However, in animal models, reperfusion at a capillary level often fails despite macrovascular recanalization – termed the “no-reflow” phenomenon. This does not reconcile particularly well with clinical experience where complete removal of thrombus generally leads to normalization of the perfusion imaging appearance (or in some cases hyperperfusion, Figure 1B), even in regions that have been irreversibly injured (“non-nutritional reperfusion”). It is possible that clinical perfusion imaging is reflecting flow in larger vessels and showing arteriolar shunting, and is too insensitive to demonstrate occlusion at the capillary level. At any rate, this phenomenon would be restricted to areas we currently regard as irreversibly injured core. To our knowledge, there has not been a description of “no-reflow” in areas thought to be penumbral prior to revascularization. Whether therapeutic strategies to prevent capillary sludging and no-reflow could transform the prognosis for regions we currently regard unsalvageable is an interesting speculation, but seems a rather distant possibility.

Tissue perfusion can also improve without recanalization due to recruitment of collateral blood flow, which can occur in some patients over time. It is visualized as a reduction in perfusion delay and improved blood flow (Figure 1A). This form of improved perfusion may be associated with clinical improvement. However, as long as the vascular occlusion remains, the patient has an ongoing risk of collateral “failure” and clinical deterioration. The mechanisms of deterioration in collateral flow are not well understood but presumably clot migration and hemodynamic fluctuations may contribute. Indeed, the observed association of general anesthesia with worse outcome after endovascular therapy (33) may relate to periprocedural hypotension impairing collateral flow. Clearly enhancing or stabilizing this retrograde collateral perfusion is a potential therapeutic strategy and has formed the basis of several attempts to improve collateral flow, although none have translated to clinical practice at this stage. Given the ongoing risk of deterioration in collateral flow, conventional anterograde reperfusion should remain the primary treatment strategy for most patients.

## Infarct Growth

The original definition of ischemic penumbra was of hypoperfused and electrically non-functional tissue that could regain function with rapid reperfusion (34, 35). This was subsequently operationalized as a tissue that was at risk of infarction in the absence of reperfusion – a somewhat different construct. Infarct growth in the absence of reperfusion is associated with worse clinical outcome and infarct growth has, therefore, been used as a surrogate outcome in trials. There are important practical considerations in the measurement of infarct growth. The aim is to measure true territorial expansion in the infarct. However, initial edema and subsequent atrophy confound this and mean that there is no perfect time to assess “growth.” In addition, progressive loss to follow-up at later time points can introduce bias as those who die and are, therefore, unevaluable are more likely to have had infarct growth. There is also uncertainty about the duration of true infarct growth, although data suggest that this is generally complete within 24 h after stroke onset (36). Assessment at 24 h is, therefore, attractive as it minimizes loss to follow-up, precedes much of the edema that peaks at 3–5 days, and can be used to assess reperfusion, recanalization, and hemorrhagic transformation.

It is important to recognize that infarct growth is not universal in the absence of reperfusion. Early follow-up imaging frequently shows regions of persistent hypoperfusion that have not developed diffusion restriction but appear to still be contributing to the observed clinical deficit (Figure 1C). The clinical significance and prognosis of persistent hypoperfusion is not well understood but it raises the possibility that infarct growth may not fully encapsulate the clinical impact of reperfusion.

## Future Directions and Ongoing Trials

There are a number of key lessons from recent trials. It seems clear that the use of non-contrast CT and clinical selection criteria will not deliver progress in extending the therapeutic time window or providing an evidence base for endovascular therapy. There are good theoretical reasons and suggestive evidence from existing neutral trials that assessing collaterals or core in addition to vessel occlusion may be beneficial and the technical requirements to achieve this are no longer an inconvenience.

Imaging selection has been hampered by a proliferation of approaches with limited standardization. The principles of identifying a target vessel occlusion and good collateral flow are well established. However, the optimal practical implementation ofthese concepts remains uncertain, and clinical practice will no doubt gravitate to the approaches that lead to success in clinical trials. Undoubtedly, the field of acute stroke therapy faces challenges but there is tremendous potential to transform clinical outcomes with new therapies, guided by imaging. It is an exciting time to be practicing stroke medicine.

## Conflict of Interest Statement

The authors declare that the research was conducted in the absence of any commercial or financial relationships that could be construed as a potential conflict of interest.
